# MFG-E8 Regulates the Immunogenic Potential of Dendritic Cells Primed with Necrotic Cell-Mediated Inflammatory Signals

**DOI:** 10.1371/journal.pone.0039607

**Published:** 2012-06-25

**Authors:** Muhammad Baghdadi, Shigeki Chiba, Tsunaki Yamashina, Hironori Yoshiyama, Masahisa Jinushi

**Affiliations:** Research Center for Infection-Associated Cancer, Institute for Genetic Medicine, Hokkaido University, Sapporo, Japan; University of Colorado School of Medicine, United States of America

## Abstract

Dendritic cells (DC) manipulate tissue homeostasis by recognizing dying cells and controlling immune functions. However, the precise mechanisms by which DC recognize different types of dying cells and devise distinct immunologic consequences remain largely obscure. Herein, we demonstrate that Milk-fat globule-EGF VIII (MFG-E8) is a critical mediator controlling DC immunogenicity in inflammatory microenvironments. MFG-E8 restrains DC-mediated uptake and recognition of necrotic cells. The MFG-E8-mediated suppression of necrotic cell uptake by DC resulted in the decreased proinflammatory cytokines production and activated signal components such as STAT3 and A20, which are critical to maintain tolerogenic properties of DC. Furthermore, the DC-derived MFG-E8 negatively regulates the cross-priming and effector functions of antigen-specific T cells upon recognition of necrotic cells. MFG-E8 deficiency enhances an ability of necrotic cell-primed DC to stimulate antitumor immune responses against established tumors. Our findings define what we believe to a novel mechanism whereby MFG-E8 regulates the immunogenicity of DC by modulating the modes of recognition of dying cells. Manipulating MFG-E8 levels in DC may serve as a useful strategy for controlling inflammatory microenvironments caused by various pathological conditions including cancer and autoimmunity.

## Introduction

Dendritic cells (DC) serve as sentinels linking between innate and adaptive responses [1]. In addition to responses triggered *via* innate immune sensing such as pathogen- and/or endogenous danger-associated signals, the ability of DCs to activate adaptive immune responses relies mainly on the processing and presentation of immunogenic antigens [2], [3]. This assumption implies that the mode of presentation and recognition of immunogenic antigens on DC may have a determinant role in the initiation and promotion of antigen-specific immune responses.

Milk-fat globule EGF-8 (MFG–E8) is a phosphatidylserine-binding protein secreted by subsets of myeloid cells that signals through engaging α_v_β_3_–_5_ integrins. The major functions of MFG-E8 are to regulate immune homeostasis through the phagocytosis of apoptotic cells [4]–[6]. We previously demonstrated that systemic targeting of MFG-E8 enhances antitumor immune responses by augmenting cross-presentation of immunogenic antigens [7]–[9]. However, how MFG-E8 directly influences the recognition systems of dying cells by DC remains largely unknown. Here we demonstrate that MFG-E8-dependent recognition of apoptotic cells facilitates the tolerogenic activity of dendritic cells, whereas necrotic cell-mediated inflammation and cross-priming of antigen-specific cells is triggered by MFG-E8-deficient DC in a RIP1 (Receptor-interacting serine-threonine kinase)-dependent manner. Thus, we delineate the novel mechanisms by which DC regulate the delicate balance between immunity and tolerance by fine-tuning recognition of dying cells in an MFG-E8-dependent manner.

## Results

### MFG-E8 maintains the tolerized phenotype of DC under steady and inflammatory conditions

**Figure 1:**
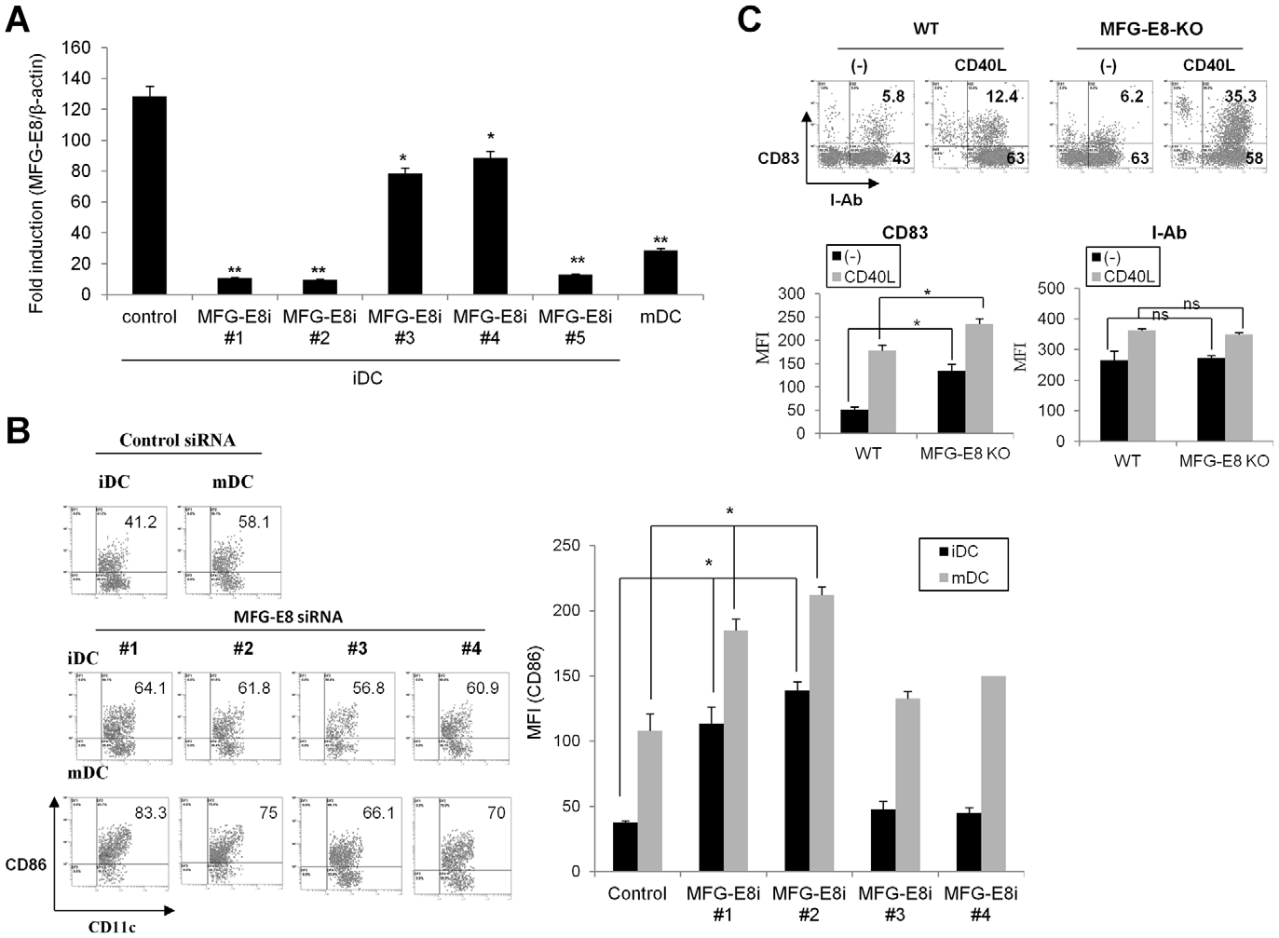
MFG-E8-deficiecy confers BMDC with an activated phenotype under steady and inflammatory conditions. (A) Five different sequences of small interfering RNA for MFG-E8 (MFG-E8i #1-#5) or a control gene (control siRNA) were introduced into immature BMDC for 48 h and the mRNA levels of MFG-E8 were quantified by RT-PCR. The mature DC (mDC) stimulated with CD40 ligand (CD40L) serve as a control due to their low MFG-E8 expression. (B) The expression levels of CD86 on CD11c^+^DC were evaluated by flow cytometry. The percentage (left) and mean fluorescence intensity (MFI) of CD86 among CD11c+DC was shown as a representative data and statistical analysis (n = 3), respectively. (C) The expression levels of CD83 and MHC-II were evaluated in DC from wild-type (WT) and MFG-E8-decifient (MFG-E8-KO) mice. The representative data (upper) and statistical analysis of three independent experiments (bottom) are shown. * *p<*0.05, ** *p*<0.01, ns: not significant.

To investigate whether MFG-E8 has impacts on the activities and immunogenicity of DC, MFG-E8 ^high^ immature DC (iDC) were generated from the bone marrow cells of wild-type or MFG-E8-deficient mice using GM-CSF. The iDC treated with an inflammatory element such as CD40 ligand served as mature DC (mDC), in which MFG-E8 levels were low [9]. In some cases, five sequences of siRNA specific for the murine MFG-E8 gene, which were validated for inhibition of murine MFG-E8 expression by RT-PCR (Figure 1A), were introduced into the wild-type BMDC. The cells were then treated with a CD40 ligand, and subjected to phenotypic analysis. Unstimulated DC from wild-type mice displayed an immature phenotype, comprising moderate levels of MHC-II, CD83 and CD86. In contrast, MFG-E8-deficient iDC or wild-type DC in which MFG-E8 gene was targeted by several siRNAs exhibited elevated expression of the costimulatory molecules CD86 and CD83 even in the absence of maturation-inducing stimulus (Figure 1B and C). Stimulation of DC with CD40L or TNF-α induced upregulation of the maturation markers CD83 and CD86, but not MHC-II at greater levels in MFG-E8-KO than wild-type DC (Figure 1B, C and data not shown). Together, these results suggest that MFG-E8 potentially restrains the co-stimulatory capabilities of DC under steady and inflammatory conditions.

### MFG-E8 deficiency facilitates the uptake of necrotic cells

**Figure 2:**
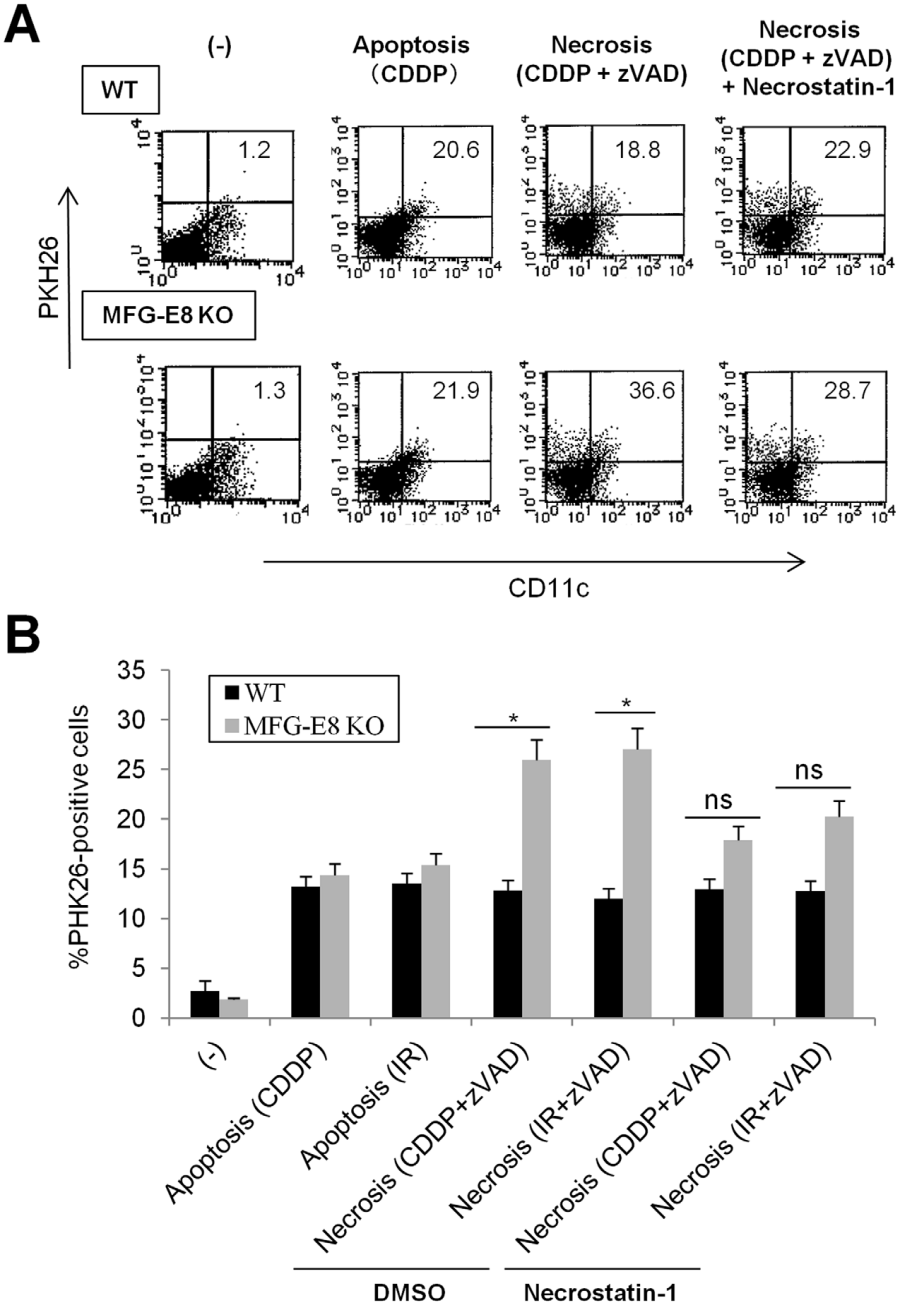
MFG-E8-deficient DC exhibit increased uptake of necrotic tumor cells. PKH26-labeled EL4 cells were treated with CDDP or γ-irradiation (IR) (30 Gy) to induce apoptotic cell death, or treated with CDDP and zVAD-fms to induce necrosis. The apoptotic or necrotic cells were incubated with BMDCs from wild-type (WT) or MFG-E8-deficient (MFG-E8-KO) mice for 2 h. In some instances, necrotic cells were treated with the RIP1 inhibitor Necrostatin-1 or DMSO for 48 h before co-culture with DC. The uptake of EL4 cells by CD11c^+^BMDCs was quantified by flow cytometry. Representative data (A) and statistical analysis from two experiments (B) are shown. The percentage of PKH26^+^CD11c^+^ and PKH26^−^CD11c^+^ populations was shown in right-upper and right-bottom quadrant of each data, respectively. Results are representative of two independent experiments. * *p<*0.05, ns: not significant.

MFG-E8 promotes the uptake and processing of apoptotic cells by DC, which may promote Foxp3^+^ regulatory T cell differentiation and suppress antigen-specific adaptive immunity [6], [9]. Several lines of evidences have revealed the impact of apoptotic cell engulfment in maintaining immune homeostasis and preventing excess inflammation [5], [10]. On the other hand, necrotic cells contribute to the formation of immunogenic microenvironments through activation of various proinflammatory mediators and danger-associated signals [11], [12]. However, whether MFG-E8-mediated recognition of necrotic cells has any impact on the effector activities of DC remains unknown. Therefore, we first evaluated the phagocytosis of dying tumor cells by MFG-E8-KO and wild-type DC. In this setting, we defined apoptotic cells as annexin-V^+^ PI^−^ cells induced by the chemotherapeutic agent cisplatin (CDDP) or γ-irradiation (IR). Necrotic cells were induced by CDDP or γ-irradiation plus the caspase inhibitor zVAD-fms, and were confirmed as Annexin-V^−^ PI^+^ populations by flow cytometry (Figure S1 and data not shown). The PKH26-labeled apoptotic or necrotic cells were loaded onto WT or MFG-E8-KO BMDC for 4 h, and measured the uptake of dying cells as CD11c^+^PKH26^+^ populations by flow cytometry. To our surprise, the ingestion of apoptotic cells was comparable between WT and MFG-E8 KO DC, indicating that BMDC may utilize compensatory machineries for apoptotic cell engulfment using other mechanisms independent of MFG-E8. In contrast, the engulfment of necrotic cells was significantly increased in MFG-E8-KO compared to wild-type DC, but was inhibited by RIP-1 inhibitor Necrostatin-1 [13] (Figure 2A and B). These results demonstrate that MFG-E8 deficiency promotes the uptake of necrotic cells in BMDC.

### MFG-E8 inhibition promotes inflammatory cytokine production in DC primed by necrotic cells

**Figure 3:**
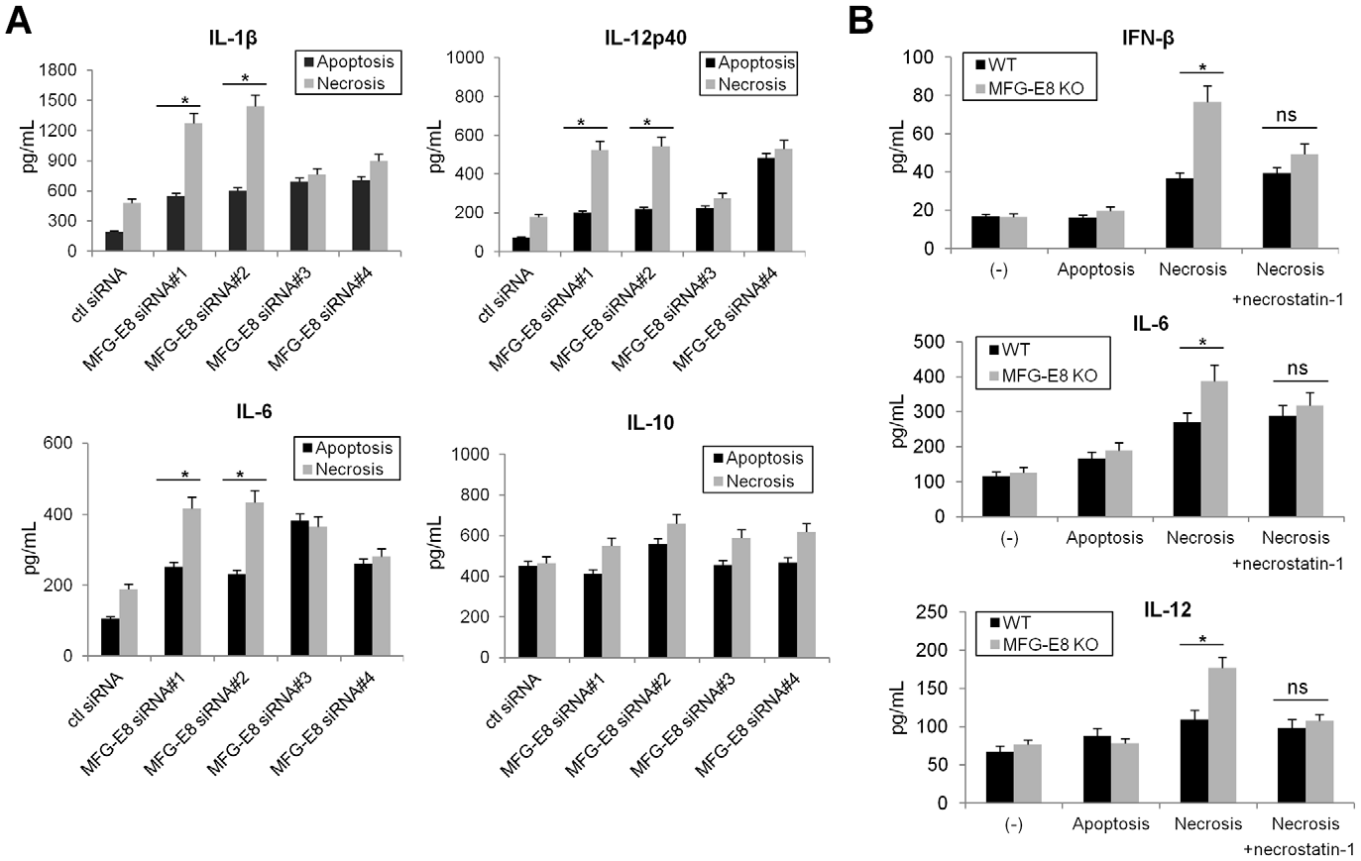
MFG-E8-deficiency increases proinflammatory cytokine production in DC. (A) BMDC transfected with control or various MFG-E8 siRNAs (#1–#4) were loaded with apoptotic or necrotic cells prepared as described in Figure 2, and the protein levels of IL-1β, IL-6, IL-12p40 and IL-10 in culture supernatants were measured by ELISA. (B) BMDC from wild-type (WT) or MFG-E8-deficient mice (MFG-E8 KO) were loaded with dying cells as described above. In some instances, necrotic cells were treated with Necrostatin-1 for 48 h before coculture with BMDC. The protein levels of IFN-β, IL-6 and IL-12 were determined by ELISA. Results are representative of three independent experiments. * *p<*0.05, ns: not significant.

We next compared the cytokine profiles of MFG-E8-KO and wild-type DC loaded with dying cells. Although MFG-E8-KO iDC had a greater capacity for production of cytokines IFN-β, IL-1β, IL-6, IL-12, but not IL-10, compared to wild-type iDC when loaded with necrotic cells, the cytokine profiles were comparable when wild-type and MFG-E8-KO DC were stimulated with apoptotic cells (Figure 3A and B). Although the MFG-E8 siRNA #3 and #4 increased IL-6 and IL-12p40 in BMDC upon ingestion of either apoptotic or necrotic cells, respectively, these might be not specific effects by MFG-E8 inhibition because these siRNA did not efficiently suppress MFG-E8 expression (Figure 1A and 3A). Moreover, the levels of pro-inflammatory cytokines were significantly suppressed in MFG-E8-KO DC when primed by Necrostatin-1-pretreated necrotic cells (Figure 3B). Together, these results demonstrate that activating necrotic signals facilitate proinflammatory cytokines production in MFG-E8-KO cell or cells in which MFG-E8 expression is inhibited by siRNA compared to MFG-E8-competent DC.

### MFG-E8-mediated uptake of necrotic cells regulates distinct sets of signals that are critical for DC homeostasis

**Figure 4:**
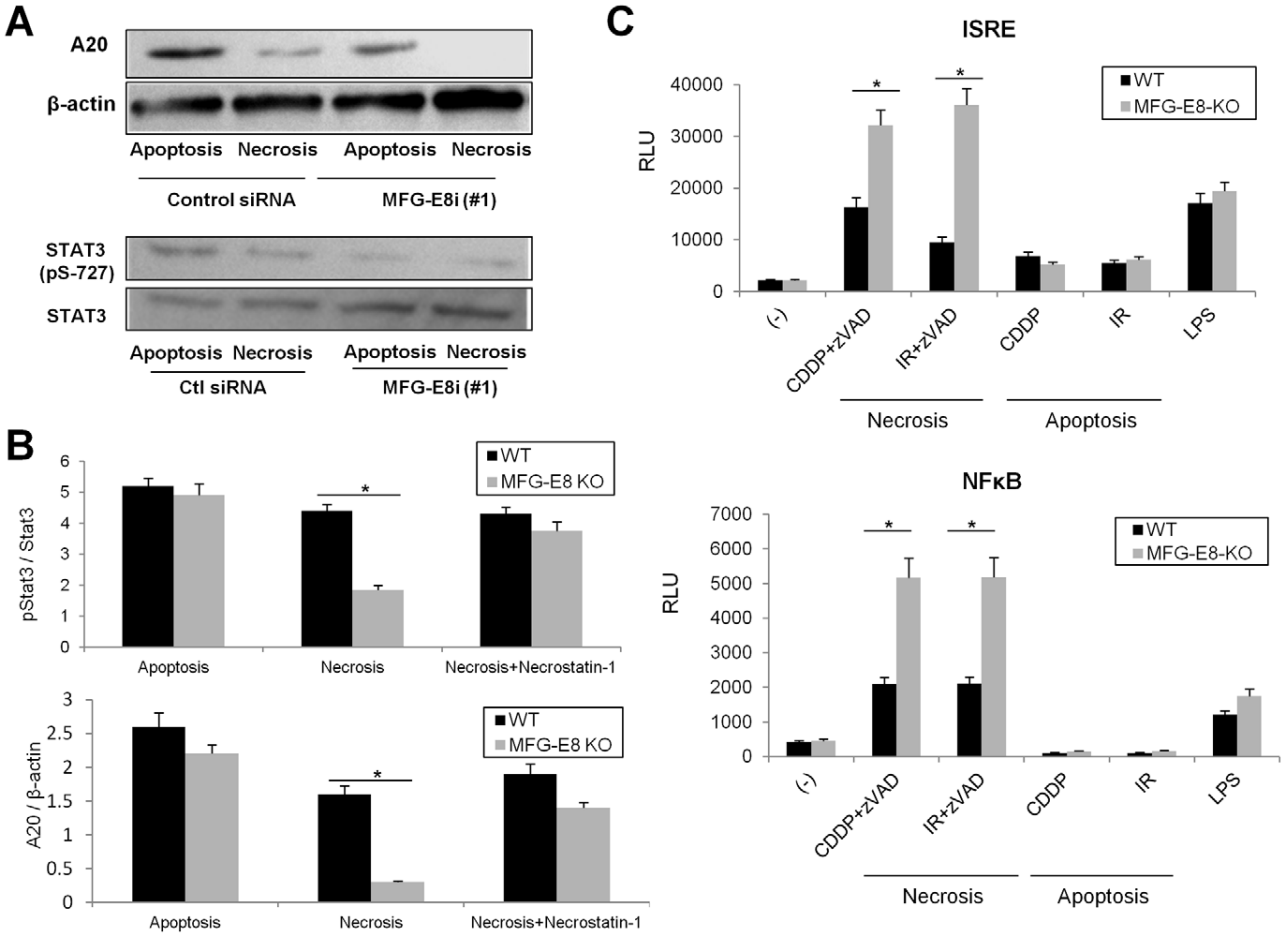
MFG-E8-mediated phagocytosis of necrotic tumor cells modulates distinct sets of signals in DC, which are critical for the maintenance of immune homeostasis. (A) Immunoblot for A20, pSTAT3 and STAT3 on lysates from BMDC transfected with control or MFG-E8 siRNA 2 h after uptake of apoptotic or necrotic cells. (B) Densitometric analysis of A20 relative to β-actin (mRNA) or p-STAT3-positive populations relative to total STAT3 (protein) in wild-type (WT) or MFG-E8-deficient (MFG-E8 KO) BMDC primed with apoptotic cells, necrotic cells or necrotic cells pretreated with Necrostatin-1 was examined by quantitative PCR or western blot, respectively. (C) WT or MFG-E8-KO BMDC were transfected with firefly luciferase ISRE or NF-κB reporter plasmids for 16 h, and loaded with apoptotic cells (CDDP and IR), necrotic cells (CDDP + zVAD-fms and IR + zVAD-fms) or LPS for 2 h. Luciferase assays were performed to measure transcriptional activities of ISRE and NF-κB. Data are representative of three independent experiments. * *p<*0.05, ns: not significant.

Accumulating evidence has validated the contribution of several sets of transcription factors to the regulation of tolerogenic DC activities [14]. In particular, the Stats and NF-κB families have emerged as critical regulators of DC immunogenic activities [15], [16]. We therefore investigated the role of MFG-E8 in the regulation of these transcription factors. Knockdown of MFG-E8 with siRNAs or MFG-E8-deficiency diminished phosphorylation levels of Stat3 in DC primed with necrotic cells, which was restored by the treatment with Necrostatin-1 (Figure 4A and B). These findings are in accord with the contribution of Stat3 to the tolerogenic functions of DC [15], [17]. Furthermore, the expression levels of the deubiquitinase A20 were markedly repressed in MFG-E8-KO DC primed with necrotic cells, which was reversed by Necrostatin-1 (Figure 4A and B). These findings were consistent with previous studies that A20 restricts proinflammatory activities and maintains the quiescent status of DC under steady and inflammatory conditions [18]. Moreover, the transcriptional activities of ISRE and NF-κB were increased in MFG-E8-KO DC primed with necrotic cells (Figure 4C). Altogether these results indicate that MFG-E8 plays an important role in the regulation of signal cascades that are critical in maintaining immune homeostasis of DC.

### MFG-E8 inhibition augments antigen-specific responses by necrotic cell-primed DC

**Figure 5:**
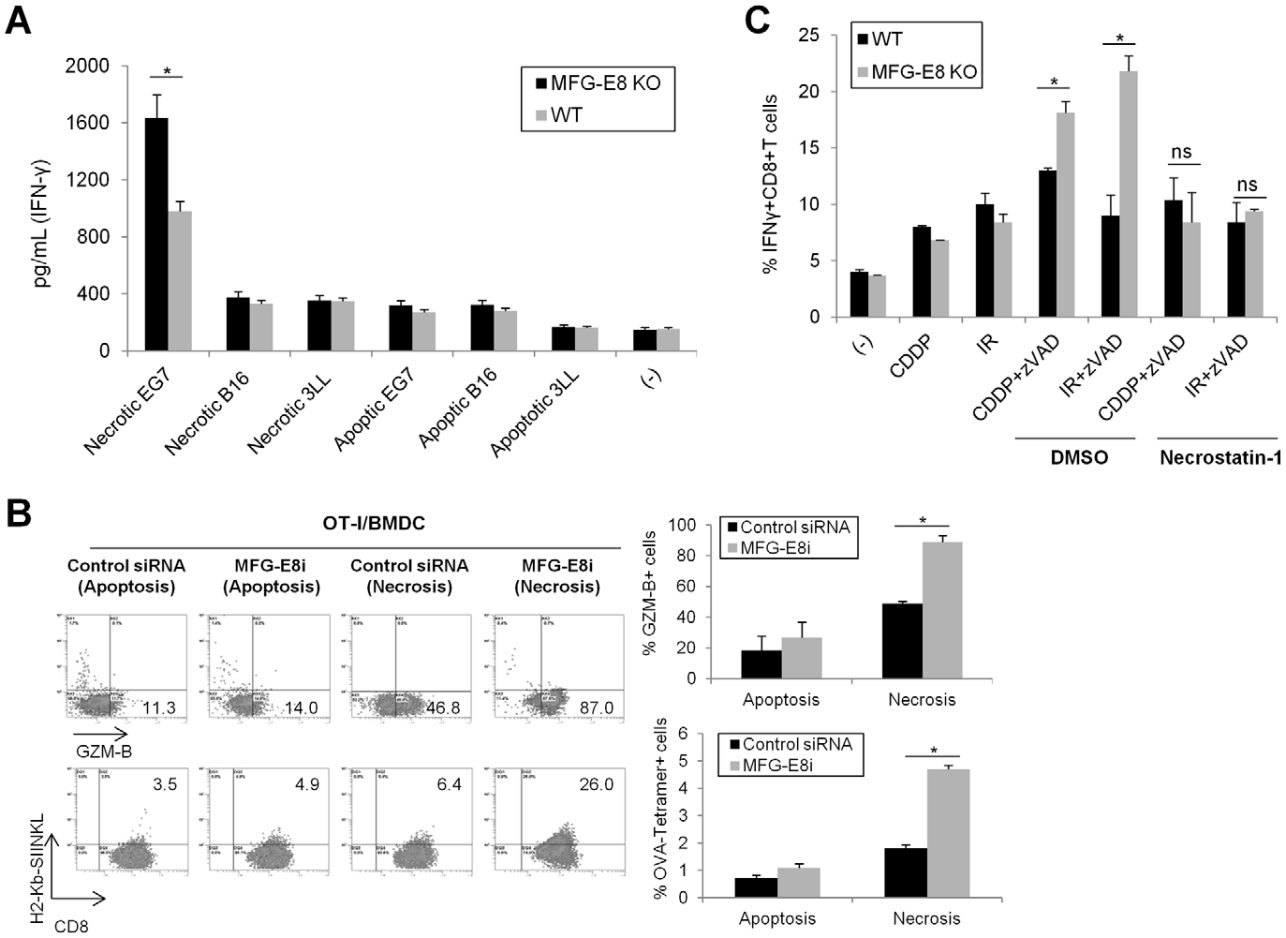
MFG-E8 attenuates cross-priming activities of necrotic cell-derived antigens in DC. (A) WT or MFG-E8-KO BMDC were loaded with apoptotic or necrotic tumor cells (EG7, B16 or 3LL) for 2 h, and co-cultured with OVA-specific TCR-transgenic CD8^+^ T cells for 72 h. IFNγ production in culture supernatants were quantified by ELISA (B) Control or MFG-E8 siRNA-transfected BMDC were loaded with EG7 cells treated with CDDP (apoptosis) or CDDP and zVAD-fms (Necrosis) for 48 h. Intracellular Granzyme-B (GZM-B) expression or H-2Kb-restricted OVA tetramer (SIINFEKL)-positive populations in CD8^+^ cells were analyzed by flow cytometry. The representative data (left) and statistical analysis of three independent experiments (right) are shown. (C) WT or MFG-E8-KO BMDC were loaded with the apoptotic (CDDP or IR) or necrotic (CDDP + zVAD-fms or IR + zVAD-fms) EG7 cells and co-cultured with OVA-specific TCR- transgenic CD8^+^ T cells to measure IFNγ production by flow cytometry. For some instances, necrotic EG7 cells were treated with Necrostatin-1 or DMSO for 12 h. Similar results were obtained from three experiments. * *p<*0.05, ns: not significant.

We next evaluated the role of MFG-E8 in the regulation of DC cross-presentation to CD8^+^ T lymphocytes. In this assay, DC were pulsed with dying tumor cells and used to stimulate purified donor CD8^+^ T cells isolated from OT-I mice, and OVA-specific IFN-γ production in the cocultured cells was assessed by ELISA. OT-I cells demonstrated robust OVA-dependent responses to cross-presentation of necrotic B16 tumor cells by wild-type DC. In contrast, minimal reactivity was induced against apoptotic EG7 cells or irrelevant apoptotic or necrotic tumor cells (B16 and 3LL) (Figure 5A). Moreover, MFG-E8-deficient DC induced a much greater potential for Granzyme-B expression and more abundant OVA-specific CD8^+^T cell populations compared to DC obtained from wild-type mice, as quantified as frequencies of H-2Kb-restricted OVA tetramer (SIINFEKL)-positive OT-I cells (Figure 5B).

Programmed necrosis mediates the activation of RIP1/3 kinase and deubiquitinating enzyme CYLD, which is counteracted by caspase-8 or FADD (Fas-associated protein with death domain) [19]–[22]. Therefore, DC pulsed with necrotic tumor cells were cocultured with OT-I cells in the presence of Necrostatin-1. The treatment of necrotic tumor cells with Necrostatin-1 prevented DC of either wild-type or MFG-E8-KO mice from activating OVA-specific CTL effector functions (Figure 5C).

These results validate that efficient cross-priming of antigen-specific T cells by DC is correlated with the extent of necrotic cell recognition, which is further enhanced by deficiency or pharmacological inhibition of MFG-E8.

### MFG-E8 is responsible for impeding the antitumor responses of DC vaccines

Although DC are potent adjuvants for triggering antitumor immunity, the clinical effects of dendritic cell-based vaccination remain unsatisfactory [1], [23]. To examine whether MFG-E8-mediated regulation of DC immunogenicity impede antitumor immunogenicity, we utilized two models for assessing in vivo anti-tumor responses by DC vaccines.

**Figure 6:**
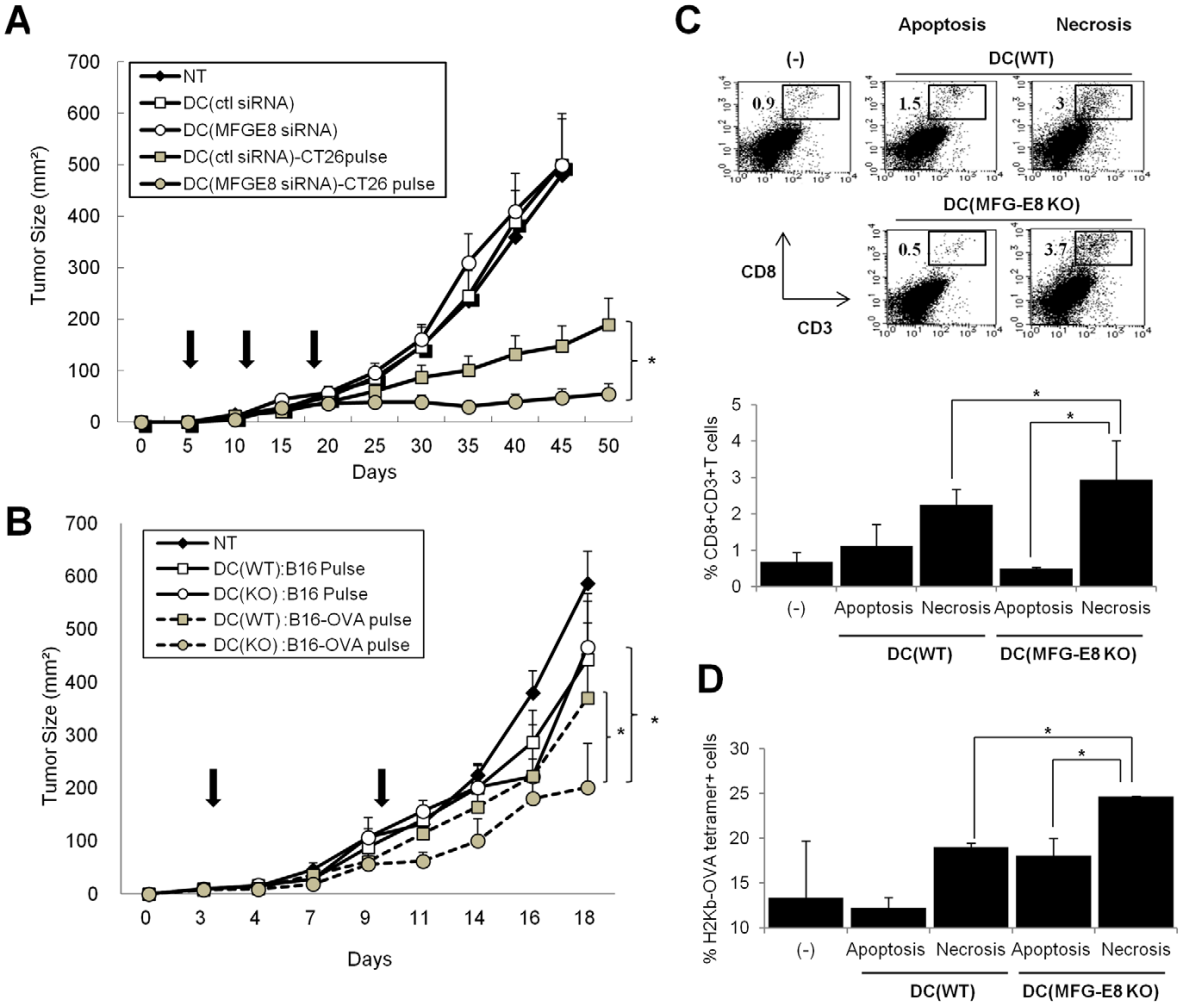
MFG-E8 inhibition stimulates antitumor adaptive immunity by necrotic cell-pulsed DC. (A) Balb/c mice were immunized with irradiated CT26 colon cancer cells (1×10^6^/mice) once a week for three times. The immunized mice (n = 5 per each group) were injected subcutaneously with a mixture of viable CT26 cells and BMDCs transfected with control or MFG-E8 siRNA-encoded lentiviral vectors and pulsed with necrotic CT26 cells or not (1×10^6^/mice) on the indicated days (Bold arrows). Tumor growth was measured on the indicated days. (B) WT or MFG-E8-KO BMDC (n = 5 per each group) were loaded with necrotic cells derived from B16 melanoma cells (B16 pulse) or those engineered to express OVA (B16-OVA) (B16-OVA pulse), and used as vaccines against established B16-OVA tumors in C57Bl/6 mice (n = 4 per group) on the indicated day. Tumor growth was measured on the indicated days (Bold arrows). Non-treatment (NT) groups serve as a control. (C, D) WT or MFG-E8-KO BMDC were loaded with apoptotic or necrotic cells derived from B16-OVA, and used to challenge C57/Bl6 mice as described above. The CD8^+^T cells were isolated and quantified from tumor tissues 5 days after tumor inoculation, and analyzed for the frequency of total CD8^+^T cells (C) or H-2K^b^-restricted OVA tetramer (SIINFEKL)-positive populations (D). Representative dot plots (C) and statistical analysis from two experiments. (C, D) are shown. Results are representative of at least two independent experiments. * *p<*0.05, ns: not significant.

First, wild-type Balb/c mice were immunized with irradiated CT26 colon cancer cells, by which CT26-derived antigens were efficiently recognized by immune cells in the tumor antigen-irrelevant naïve mice [24]. The immunized mice were then injected subcutaneously with a mixture of viable CT26 cells and BMDC transfected with control or MFG-E8 siRNA-encoded lentiviral vectors and pulsed with necrotic CT26 cells or not. The treatment with control or MFG-E8 siRNA-transfected, unpulsed DC was ineffective to control the established tumors, whereas the vaccination of MFG-E8 siRNA-transfected DC pulsed with necrotic CT26 cells induced superior antitumor responses compared to those of control siRNA-transfected ones (Figure 6A).

As a second model, immature BMDCs generated from wild-type or MFG-E8 KO mice were loaded with necrotic B16-OVA (MO-4) or B16 cells, and then used as a vaccine adjuvant against established B16-OVA tumors. The treatment with wild-type DC primed with necrotic B16 tumor cells was ineffective against established tumors. In contrast, the vaccination of MFG-E8-KO DC loaded with necrotic B16-OVA cells triggered substantial antitumor effects at greater levels than those of wild-type DC loaded with necrotic B16-OVA cells (Figure 6B). The vaccination with the MFG-E8-KO DC did not different from those with the WT DC on antitumor effects when DC were loaded with apoptotic B16-OVA cells (data not shown).

These results indicate that pre-immunization or presence of tumor-specific antigens might be required for breaking tolerance, but MFG-E8 inhibition or deficiency enables DC vaccines to further promote tumor eradication in the *in vivo* settings.

To analyze the mechanisms underlying these effects, we isolated tumor tissues derived from B16-OVA from mice with established tumors after treatment with the DC vaccines primed with necrotic or apoptotic B16-OVA tumor cells. Vaccination with necrotic cell-primed MFG-E8-KO DC produced greater frequencies of CD8^+^ T cells in tumor-infiltrating lymphocytes isolated from mice than did necrotic cell-primed WT DC (Figure 6C). Quantification of tumor-infiltrating CD8^+^ T cells expressing Granzyme-B or binding H-2K^b^-OVA tetramer revealed that vaccination with necrotic cell-primed MFG-E8 KO DC increased the frequency of OVA-specific CD8^+^ cytotoxic T lymphocytes in tumor tissues (Figure 6D and Figure S2). In contrast, the CTL frequencies and activities in spleen from MFG-E8-deficient mice were comparable to those from wild-type mice (data not shown).

Collectively, these findings validate the importance of MFG-E8 in attenuating protective antitumor immunity by fine-tuning the recognition of signals delivered by necrotic tumor cells.

## Discussion

Recent evidence has unveiled the significance of distinct types of cell death signals in the regulation of immune homeostasis, developments, and effector activities [5], [10], [25]. In particular, non-apoptotic types of cell death, represented by autophagy and necrosis, have emerged as alternative pathways in the modulation of inflammation and protective immune responses [12]. In fact, necrotic cell death mediates inflammation and autoimmunity through TNFR and RIP1/3-mediated antagonism of apoptotic pathways [19]–[22]. However, few mediators that differentially recognize distinct cell death and are suitable for therapeutic manipulation have been identified.

We demonstrate in this study that MFG-E8 functions as an endogenous effector of DC immunogenicity, and attenuates pro-inflammatory and antitumor immunity through suppression of DC cross-presentation of necrotic cell-derived antigens. These findings validate an unexpected function of MFG-E8 in dictating the immune recognition systems, which may be dependent on distinct types of cell death signals.

MFG-E8 regulates DC immunogenicity by manipulating discrete sets of downstream signals that are critical for the maintenance of immune homeostasis, including Stat-3 and A20. Previous studies suggested that MFG-E8 activates diverse array of oncogenic signals such as Stat3, Hedgehog, Akt/PI3K and twist-1, in tumor cells [7], [26]. Although MFG-E8 serves as ligand for integrin-αvβ3, it remains largely unknown whether MFG-E8 controls such a broad array of cascades in immune and tumor cells through integrin-mediated signals. Further studies should identify downstream adaptors of MFG-E8 that convey oncogenic and tolerogenic signals by integrin-dependent and –independent mechanisms.

MFG-E8 may affect the quality and magnitudes of antigen-specific responses by modulating apoptotic cell engulfment and processing [4], [9]. We showed that MFG-E8 restrains the cross-priming of necrotic cell-derived antigens by DC, but cross-presentation of target antigens *via* apoptotic cell recognition is ineffective irrespective of MFG-E8-mediated phagocytosis. Recent evidence reveals that the death signals related to apoptosis, such as caspase-8 and FADD, serve as negative regulators of necroptosis by suppressing RIP3 kinase-dependent signals [27]–[29]. In this respect, it is tempting to speculate that MFG-E8-mediated signals may interact with sentinels of apoptotic death signals, such as FADD and pro-apoptotic Bcl–2 family members. Alternatively, the co-factors and adaptors activated by MFG-E8 may interfere with the TNF-mediated inflammatory signal cascade and RIP kinase activities in dying tumor cells. In this context, it is also important to clarify the molecular linkage between necrotic-associated signals and cross-priming of particular antigens arising from dying tumor cells.

Recent evidence has clarified that efficient cross-presentation of immunogenic antigens serves as a key goal to achieve a multiple array of antitumor immune responses by DC-targeted vaccines [1], [14], [30]. Moreover, the activation of innate immune signals mediated by TLR, NLR and/or CD40 may sense DC to facilitate cross-presentation of immunogenic tumor antigens and trigger specific T cell responses [31]. In this regard, the pharmacological targeting of MFG-E8 in combination with other sets of innate immune adjuvants may has a profound impact on exploring new type of DC-based immunotherapy, which may lead to efficient control of advanced tumors.

In summary, our findings provide novel mechanisms by which targeting MFG-E8 may generate potent antitumor modalities by facilitating recognition of immunogenic antigens and suppressing deleterious processing of dying cells. The coupling of MFG-E8 inhibitors to agents that stimulate necrotic tumor cell death might be considered a new strategy for improving current anticancer regimens in clinical settings.

## Materials and Methods

### Mice

C57BL/6 and Balb/c mice were purchased from SCL and Charles River, respectively. MFG-E8 knockout mice and OT-I mice were kindly provided by Prof. Shigekazu Nagata (Kyoto University) and Prof. Shigeo Koyasu (Keio University), respectively. All experiments were conducted under a protocol approved by the animal care committees of Hokkaido University.

### Tumor cells

The tumor cells (CT26 colon carcinoma cells, B16 melanoma cells, and EG7 thymoma cells) were obtained from the American Tissue Culture Collection (ATCC). B16 melanoma cells expressing OVA genes (B16-OVA: clone MO4) were kindly provided by Professor Hideo Yagita (Juntendo University) and used as described previously [8]. All cell lines described above were obtained one year before experiments were initiated, and authenticated by the Central Institute for Experimental Animals (Kawasaki, Japan) for interspecies and mycoplasma contamination by PCR three months before the experiments.

### siRNA construction and transduction

The design, preparation, and transduction of siRNA vectors were performed as described previously [7]. In brief, expression plasmids containing murine MFG-E8 siRNA were obtained from Invitrogen, and transfected into DC according to the manufacturer's instructions. The gene knockdown efficacies were assessed by RT-PCR and were ranged from 35 to 95% (Figure 1A). The siRNA sequences specific for murine MFG-E8 were:

5′-CCGAGACCAACTACTACAACCTGGA-3′ (#1); 5′-CCAACTACTACAACCTGGATGGAGA-3′ (#2); 5′-CCTCCAGCAGCTACAAGACATGGAA-3″(#3); 5′-CCCACTTGGGAAGGCTGGATAATCA-3′ (#4); 5′-CACATCCAGTATGTGGCGTCCTACA-3′ (#5).

### DC phenotype

The phenotype of BMDC was analyzed by flow cytometry using anti-CD11c, anti-CD83, anti-CD86 and anti-MHC-II (BD Bioscience). For intracellular staining, BMDC were treated with brefeldin-A (Sigma-Aldrich), stained with anti-CD11c, fixed, permeabilized with Cytofix/Cytoperm buffer, and stained again with PE-conjugated Abs for phospho-Stat3 (BD Bioscience). In some cases, IFN-γ, IL-10 and IL-12 were quantified by ELISA (BD Bioscience) using supernatant obtained from cultured BMDC.

### Phagocytosis assay

B16 melanoma cells were treated with CDDP or exposed to γ-irradiation (30 Gy) to trigger apoptosis (more than 80%). Other cells were treated with CDDP or γ-irradiation in the presence of caspase inhibitor zVAD-fms or subjected to multiple cycles of freeze-thaw to trigger necrosis. The apoptotic or necrotic cells were labeled with PKH26 red fluorescent dye (Sigma-Aldrich). BMDCs were cocultured with the labeled apoptotic or necrotic cells for 4 h and phagocytosis efficiency evaluated by flow cytometry.

### Immunoblotting

BMDC transfected with control or MFG-E8 siRNA were subjected to Western blotting using antibodies against phospho-Stat3, Stat3, A20 (all from Cell Signaling Technology).Beta-actin was used as a loading control to check the integrity of each sample. The densitometries of Stat-3, phospho-Stat-3 or A20 were quantified using image J software.

### Transcriptional activities of ISRE and NF-kB

The transcriptional activities of DC were assayed as described previously [32]. In brief, BMDC were transfected with firefly luciferase ISRE (Interferon-stimulated response element) or NF-κB reporter plasmids and 16 h later, loaded with apoptotic or necrotic cells for 2 h. Luciferase assays were performed to measure the transcriptional activities of ISRE and NF-κB according to manufacturer's instructions (Promega).

### In vitro cross-priming assay

BMDCs were co-cultured in 96-well round-bottom plates for 4 h with apoptotic or necrotic B16-OVA or antigen-irrelevant B16 or EL-4 cells (1∶10 ratio) that had been labeled with PKH26. CD11c^+^ cells were isolated by magnetic cell sorting (Miltenyi Biotec), and cocultured with naïve CD8^+^ T cells from the spleens of OT-I mice for 72 h. Intracellular IFNγ or Granzyme-B expression in T cells was then determined by flow cytometry. In some experiments, the tumor cells were pretreated with Necrostatin-1 (100 μM) before the co-culture to evaluate the contribution of necrotic cell uptake to the cross-priming of OVA-specific CTLs.

### In vivo antitumor effects of DC vaccines

For in vivo tumor experiments, Balb/c mice were immunized with irradiated CT26 colon cancer cells (1×10^6^/mouse) once a week for three times. The immunized mice were injected subcutaneously with a mixture of viable CT26 cells and BMDCs transfected with control or MFG-E8 siRNA-encoded lentiviral vectors and pulsed with necrotic CT26 cells or not (1×10^6^/mouse) on day 7 and 14 after tumor inoculation. In additional experiments, C57BL/6 mice were challenged subcutaneously in the flank with B16 melanoma cells on day 0. For the therapy model, intratumoral administration of wild-type or MFG-E8-KO DC (1×10^6^/mouse) pulsed with necrotic B16 or B16-OVA cells were performed once a week for two times. Tumor growth was measured every three days.

### Phenotypic analysis of tumor-infiltrating CD8^+^ T cells

Tumor infiltrating lymphocytes were obtained from B16-OVA challenge sites using a Nocoprep (Axis-Shield) cell gradient separation. The frequencies of CD8^+^T cells were analyzed by flow cytometry using mAb against CD8 and CD3e. The frequencies of OVA specific CTL were determined by staining tetramers recognizing H2Db-restricted OVA sequence (SIINFEKL).

### Statistics

The differences between two groups were determined with the Student's *t* test or the two-sample *t* test with Welch's correction. The differences among three or more groups were determined with a one-way ANOVA. The *P* values less than 0.05 are considered statistically significant. * *p<*0.05, ** *p*<0.01, ns: not significant.

## Supporting Information

Figure S1 **Necrostatin-1 suppresses necrotic cell death mediated by CDDP and zVAD-fms.** EL4 thymoma cells were treated with chemotherapeutic agent cisplatin (CDDP), γ-irradiation (IR) with or without pan-caspase inhibitor zVAD-fms to induce necrosis and apoptosis, respectively. In some instances, RIP-1 kinase inhibitor Necrostatin-1 was added before CDDP and zVAD-fms treatment. The necrotic or apoptotic cell death was shown as annexin-V^−^/PI^+^ or annexin-V+/PI^+ or –^ populations, respectively, by flow cytometry.
(TIF)

Figure S2 **In vivo vaccination with MFG-E8-KO DC enhanced granzyme-B expression in intratumor CTL.** WT or MFG-E8-KO BMDC was loaded with apoptotic or necrotic cells derived from B16-OVA, and inoculated into B16-OVA tumors raised from C57/BL6 mice. The cells were isolated from tumor tissues 5 days after DC treatment, and analyzed for Granzyme-B expression on CD8^+^T cells by intracellular flow cytometry. Representative data (above) and statistical analysis of total experiments (n = 2) (bottom) is shown.
(TIF)
