# Supplementary figures and images for: MFG-E8 Regulates the Immunogenic Potential of Dendritic Cells Primed with Necrotic Cell-Mediated Inflammatory Signals
Source: PLoS One. 2012 Jun 25;7(6):e39607. doi: 10.1371/journal.pone.0039607 (PMC3382463; doi:10.1371/journal.pone.0039607)

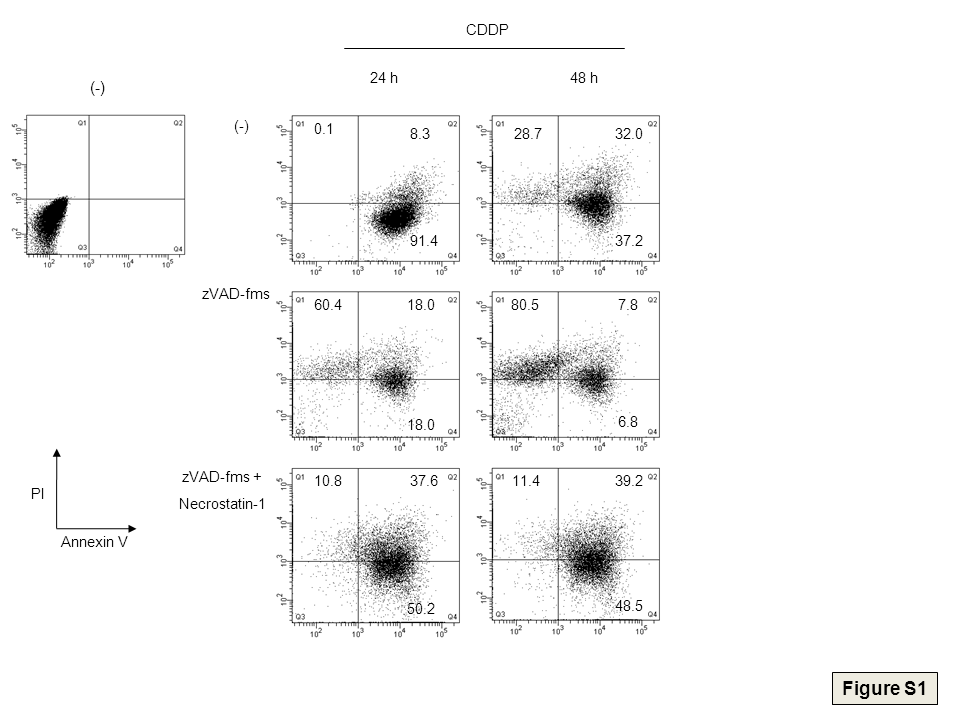

Supplement: Figure S1 — Necrostatin-1 suppresses necrotic cell death mediated by CDDP and zVAD-fms. EL4 thymoma cells were treated with chemotherapeutic agent cisplatin (CDDP), γ-irradiation (IR) with or without pan-caspase inhibitor zVAD-fms to induce necrosis and apoptosis, respectively. In some instances, RIP-1 kinase inhibitor Necrostatin-1 was added before CDDP and zVAD-fms treatment. The necrotic or apoptotic cell death was shown as annexin-V−/PI+ or annexin-V+/PI+ or – populations, respectively, by flow cytometry. (TIF) [file pone.0039607.s001.tif]

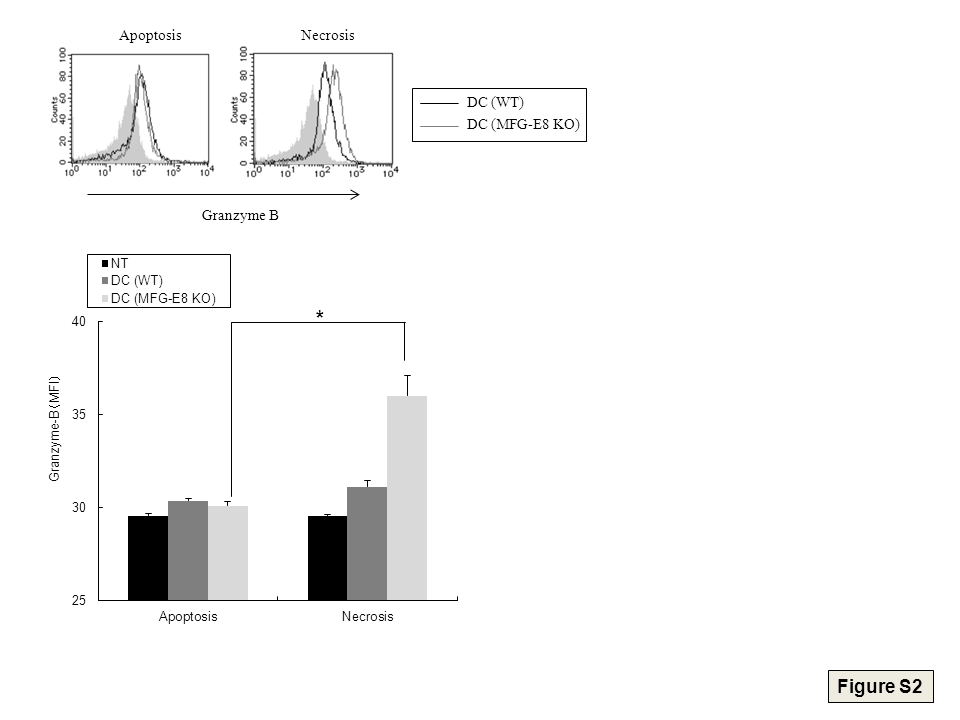

Supplement: Figure S2 — In vivo vaccination with MFG-E8-KO DC enhanced granzyme-B expression in intratumor CTL. WT or MFG-E8-KO BMDC was loaded with apoptotic or necrotic cells derived from B16-OVA, and inoculated into B16-OVA tumors raised from C57/BL6 mice. The cells were isolated from tumor tissues 5 days after DC treatment, and analyzed for Granzyme-B expression on CD8+T cells by intracellular flow cytometry. Representative data (above) and statistical analysis of total experiments (n = 2) (bottom) is shown. (TIF) [file pone.0039607.s002.tif]
